# The Gaza health information system: Rebuilding for a resilient future

**DOI:** 10.1371/journal.pgph.0004700

**Published:** 2025-06-16

**Authors:** Nima Yaghmaei, Ente Rood, Amal Arafeh, Zaher Whaidi, Wafa’a Zeidan, Paul B. Spiegel, Amrish Y. Baidjoe

**Affiliations:** 1 KIT Royal Tropical Institute, Amsterdam, the Netherlands; 2 Department of Health, UNRWA, Amman, Jordan; 3 Palestinian Health Information Center, Ministry of Health, Gaza, Palestine; 4 Center for Humanitarian Health, Department of International Health, Johns Hopkins Bloomberg School of Public Health, Baltimore, Maryland, United States of America; 5 Luxembourg Operational Research and Epidemiology Support Unit, Médecins Sans Frontières, Luxembourg City, Luxembourg; 6 Médecins Sans Frontières, Operational Centre Brussels, Brussels, Belgium; PLOS: Public Library of Science, UNITED STATES OF AMERICA

The ongoing Israel-Gaza war has led to the rampant destruction and disruption of much of Gaza’s health system, including the deliberate targeting of civilian infrastructure and the loss of access to essential medical services [1,2]. These losses include the critical infrastructure and capacity for Gaza’s once-robust Health Information System (HIS). The disruption to the HIS has greatly challenged decision-making, health service delivery, humanitarian response, and data driven advocacy.

Before October 2023, Gaza boasted a particularly robust health information system considering the circumstances, serving as a potential model for similar contexts. Despite some fragmentation, the former HIS covered a wide range of public health functions, including medical records, hospital functionality, primary health services, human resources, supply chains and financial systems, as well as numerous population health indicators and active disease surveillance.

## Current state of the HIS

The ongoing conflict in Gaza has severely disrupted the functioning of the different HIS, including the destruction of critical supporting infrastructure, leading to the loss of public health data and medical records [3]. The clearest example is the early 2024 destruction of Al-Shifa Hospital, which hosted the main data center for national health information [4]. This regression has created significant challenges in assessing the impacts of the ongoing military siege. Furthermore, the instability of electricity supply and internet connectivity has further complicated these efforts.

The constant displacement of the population in Gaza, which by early 2025 included approximately 90% of the civilian population or 1.9 million people, has made it extremely difficult to maintain an accurate view of the populations needs [2]. Health surveillance, morbidity, mortality, and service utilization data are crucial to calculate health care and resource needs [5]. However, these insights are difficult to achieve with the current level of displacement and active conflict. In addition, healthcare workers, many of whom are already overburdened, now face the additional task of manually recording patient data, merging and cleaning datasets, and uploading to data when possible. In addition to the overburdening of healthcare workers by increases in health service demand, healthcare workers are themselves traumatized by the systematic attacks on their peers [6,7]. Therefore, relying on frontline healthcare workers to record and maintain a decentralized health information system in the current situation in hardly feasible.

Despite these challenges, organizations are still maintaining various forms of health information. The Ministry of Health is currently employing surveillance systems for mortality, outbreaks, referrals and trauma, while the UN Relief and Works Agency (UNRWA) is currently recording, and reporting via dashboard, data on nutrition, water access, hygiene, sanitation, disease surveillance, and facility monitoring.

## Rebuilding a more resilient and cohesive system

**Fig 1:**
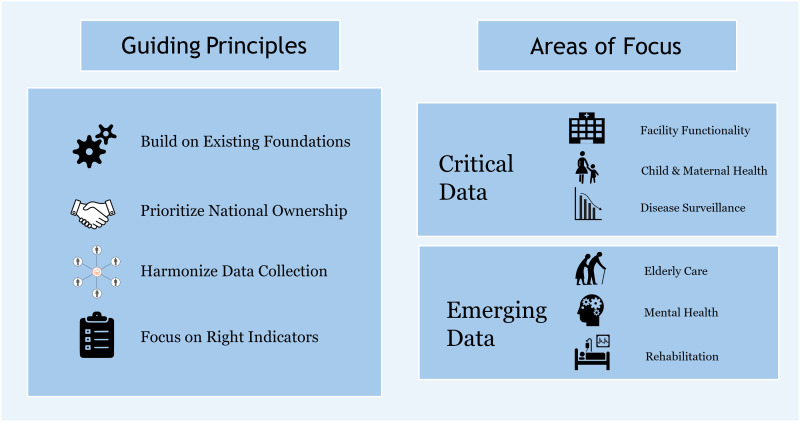
Guiding principles and key areas of focus for rebuilding Gaza’s health information system.

As we look towards rebuilding Gaza’s health information system, it is imperative to create an even more resilient and cohesive system. Fig 1 presents the key principles by which the rebuilding effort should be guided:

1 **Build on existing foundations**: Despite the extensive disruption, elements of the previously strong system remain. These elements, such as existing human capital, and established system design and reporting channels, must be identified and utilized.
2 **Prioritize national ownership**: The rebuilding process should be led by local health authorities, with international organizations playing a supportive role through technical expertise and funding [8]. This was demonstrated during the successful 2024 Gaza polio vaccination program which was led by the Palestinian Ministry of Health with support from multilateral organizations [9].
3 **Harmonize data collection:** A unified, centralized system compatible across organizations is crucial to avoid fragmentation and duplication [8]. Prior to 2023, the Gaza health system struggled with significant fragmentation, which in turn affected the health information system [10]. Success in harmonization has been observed in Lebanon where primary health data on Palestinian refugees is centralized in a refugee health database to help deliver targeted health services [8].
4 **Focus on the most appropriate indicators at the right time**: We must balance our “data hunger” tendencies with the realities of limited resources and overburdened staff [11–13].

## Key areas of focus for data collection

As we rebuild the HIS, we must focus on six critical and emerging areas, as determined by the HIS session of the 3^rd^ Gaza Health Initiative Conference [14].

### Critical areas

1 **Child and maternal health:** Tracking indicators related to child and maternal health are crucial, given the vulnerability of these populations.
2 **Facility functionality**: Monitoring the operational status and capacity of health facilities and ancillary services is essential for effective resource allocation and service delivery. Fluctuations in service capacity across the health system are ongoing, driven by the stop-start nature of ceasefires and humanitarian access [2].
3 **Disease surveillance**: Robust surveillance systems are vital for early detection and response to potential outbreaks which may be triggered by the severe disruptions to health services or critical infrastructure. The need for disease surveillance was best demonstrated by the 2024 outbreak of polio in Gazan children [9].

### Emerging areas

4 **Elderly care:** Often overlooked in humanitarian crises, the elderly population, who have unique health needs, requires specific attention [15]. Thus, indicators specific to elderly care are needed.
5 **Mental health**: The psychological impact of prolonged conflict necessitates a dedicated focus on mental health data for the population and health workers.
6 **Physical rehabilitation**: With the high number of injuries, tracking rehabilitation needs and progress is essential for long-term health planning.

## Challenges and opportunities

Gaza’s health information system is currently facing numerous challenges. However, there is an opportunity to rebuild using key guiding principles to increase resilience. Community-based surveillance could play a crucial role in early disease detection and response, as demonstrated by the rapid response to the 2024 polio outbreak [9]. Creative use of proxy indicators, such as food distribution data or vaccination records, could help estimate highly mobile populations in the absence of accurate census data [8]. The use of cloud servers and mobile applications could be expanded to enhance data collection and accessibility. Collaboration between various actors – such as the Ministry of Health, UNRWA, WHO, and other NGOs can establish a harmonized and efficient information system, allowing for a coordinated effort to collect, manage, and disseminate interoperable and standardized data. Finally, a resilient health information system will allow for documentation of the health and well-being of a population under siege and contribute to constituting historical accountability.

## Conclusion

Medical and public health data is essential, particularly within complex emergencies such as active conflict. It informs the local and international understanding of the scope and scale of health impacts, and adds essential information for global advocacy beyond the medical sphere. Rebuilding Gaza’s health information system should not just focus on lost infrastructure, but instead on creating a more resilient, integrated, and responsive system. By building on existing foundations, including the local national capacity, prioritizing national ownership, harmonizing data collection, and focusing on the critical and emerging indicators, a robust health information system will be developed which can further serve as a model for other conflict-affected settings, and hopefully one day to a hopeful path of recovery.
